# An Approximately Isotropic Origami Honeycomb Structure and Its Energy Absorption Behaviors

**DOI:** 10.3390/ma16041571

**Published:** 2023-02-13

**Authors:** Jiayue Zhai, Dingguo Zhang, Meng Li, Chengbo Cui, Jianguo Cai

**Affiliations:** 1 School of Sciences, Nanjing University of Science and Technology, Xiaolingwei Street No. 200, Nanjing 201194, China; 2 Qian Xuesen Laboratory of Space Technology, China Academy of Space Technology, Youyi Street No. 104, Haidian, Beijing 100094, China; 3 Key Laboratory of C & PC Structures of Ministry of Education, National Prestress Engineering Research Center, Southeast University, Nanjing 211189, China

**Keywords:** origami metamaterial, honeycomb materials, approximately isotropic, response surface methodology

## Abstract

Honeycomb structures have a wide range of applications owing to their light weight and promising energy absorption features. However, a conventional honeycomb structure is designed to absorb impact energy only in the out-of-plane direction and demonstrates unsatisfactory performance when the impact energy originates from a different direction. In this study, we proposed an origami honeycomb structure with the aim of providing an approximately isotropic energy absorption performance. The structure was created by folding a conventional honeycomb structure based on the Miura origami pattern, and it was investigated using both numerical and experimental approaches. Investigations of the structural behaviors under both out-of-plane and in-plane compressions were conducted, and the results revealed significantly different deformation modes in comparison with those of a conventional honeycomb structure. To determine the influences of geometries, we conducted a series of numerical studies, considering various structural parameters, and analyzed the response surface of the mean stress in three directions. Based on the numerical and experimental results, a parameter indicating the approximate isotropy of the origami honeycomb structure was introduced. The proposed structure is promising for absorbing energy from any direction and has potential applications in future metamaterial design work.

## 1. Introduction

Cellular structures are considered to be the most promising structures for energy absorption and structural protection owing to their low density, low stiffness, controllable deformation, and mature processing technology [1,2,3,4,5,6,7]. Cellular structures have excellent characteristics within a certain range or in a specific direction, whereas in other directions, they inevitably demonstrate limitations in their mechanical properties. For example, owing to the particularity of honeycomb structures, their in-plane strength is merely 1/20 of their out-of-plane strength [4,8,9,10]. Therefore, when honeycomb structures are used as energy-absorbing structures, they typically absorb energy only in the out-of-plane direction of the cells. To improve the specific energy absorption, most honeycomb structural designs are focused on improving the buckling deformation capacity [11,12,13,14,15]; however, this does not solve the problem of the significant difference between the strengths in the in-plane and out-of-plane directions.

Mechanical metamaterials are man-made structures with counterintuitive mechanical properties [16]. In comparison with conventional materials, metamaterials exhibit various significantly improved mechanical properties, which are typically associated with four elastic constants: Young’s modulus, the shear modulus, the bulk modulus, and Poisson’s ratio [17]. The particularity of this mechanical performance is due to the geometric characteristics of the structure rather than the material parameters, such as zero or negative Poisson’s ratios [18,19], tunable stiffness [20], negative compressibility [21], and thermal [22] characteristics. In recent years, the origami structure has been widely used as a metamaterial in the engineering field [23,24,25,26]. The creases in the origami structure are similar to the “initial geometric defects”, which can spread external forces and accelerate the spread of structural deformation to the undeformed area, thereby dissipating energy and improving the mechanical properties of the buffer structure [14,27,28,29,30,31,32]. Zhang et al. [30] constructed two types of patterns by introducing basic pyramid elements on the surface of thin-walled square tubes. The numerical results indicate that the square tube undergoes an octagonal collapse mode during the axial compression process. This collapse mode effectively reduces the initial peak force and significantly improves the energy absorption capacity. Ma et al. [33] conducted experimental studies on the square tubes with pyramid patterns, and no complete octagonal mode was observed in the axial crushing tests. The test results indicate that the octagonal mode is a desirable collapse mode; however, it is unstable. To address this problem, You et al. [34,35] proposed an “Origami crash box” functional unit, which introduces creases at the corners of the square tubes. This unit can produce a completed diamond mode and produce additional plastic hinge lines. Hence, the energy absorption capacity is significantly improved.

Miura origami is a typical rigid origami structure with a combination of geometric simplicity and excellent mechanical properties. This novel structure has attracted the attention of researchers, and many different aspects of the structure have been studied [36,37,38,39,40]. You et al. [37,39,41,42,43] conducted quasi-static and dynamic compression experiments on metamaterials based on the Miura origami method. The research shows that the specific energy absorption of the structure is better than that of the conventional honeycomb with the identical relative density within a certain range of geometric parameters. It was also demonstrated experimentally and numerically that the multilayered metamaterial has periodic gradient stiffness when subjected to out-of-plane quasi-static compressive loads [43]. Compared with uniform metamaterials, origami metamaterials with gradient distribution can effectively avoid the force drop after the initial peak force, and the energy absorption capacity is significantly improved [39]. These recent studies have analyzed the mechanical properties and energy absorption performances of origami-inspired structures well and made it easier to utilize Miura-derivative origami patterns for various applications. In our earlier work, a high-performance energy-absorbing structure called pre-folded honeycomb was designed by introducing the Miura origami pattern to conventional honeycomb [44]. The folding pattern and energy absorption performance of the pre-folded structure under in-plane impact loading were investigated numerically and experimentally. The accuracy of the simulation results was verified by experiments [45]. In addition, the energy absorption characteristics of origami honeycomb during out-of-plane crushing were also theoretically derived in [46]. The error between the theoretical and simulation results was between −8.55% and 6.50%. Our research found that the programmable design of the in-plane and out-of-plane strength of honeycomb structure can be realized within a certain range by adjusting the crease. Further, mechanical metamaterials with specific requirements can be designed, which improve the application range of origami honeycomb. Wei et al. [47] manufactured origami aluminum honeycomb for the automobile energy absorbing box via a welding process. The analysis results show that at least 10% of the kinetic energy of the vehicle can be absorbed during the collision. It can be seen that the origami honeycomb structure has great practical value and application prospects.

On the basis of previous work, a design method for approximately isotropic origami honeycomb is proposed in this study. The approximate isotropic coefficient k was introduced to express the degree of isotropy of the origami honeycomb, and the relationship between the folding parameters of the origami honeycomb and the approximate isotropic coefficient was expressed within a certain range. The objective of the proposed approximately isotropic design is to build a foundation such that the origami honeycomb can be more easily applied to the field of energy absorption and buffer structure.

The rest of this paper is organized as follows: in Section 2, the origami honeycomb model is introduced, and the relationship between the geometric parameters is presented; Section 3 presents numerical simulations of the origami honeycomb to obtain the responses under in-plane and out-of-plane quasi-static compressions, and the analysis of the deformation modes of the origami honeycomb under compressions in different directions; in Section 4, the approximate isotropy of the origami honeycomb is defined, and the calculation formula of the approximate isotropy coefficient k is given; Section 5 presents the simulation and compression experiments conducted on the approximately isotropic origami honeycomb structure; and finally, conclusions and suggestions are provided.

## 2. Geometry of the Origami Honeycomb

**Figure 1:**
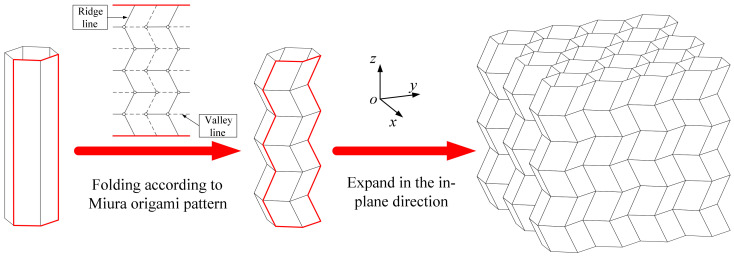
Conventional honeycomb and origami honeycomb.

**Figure 2:**
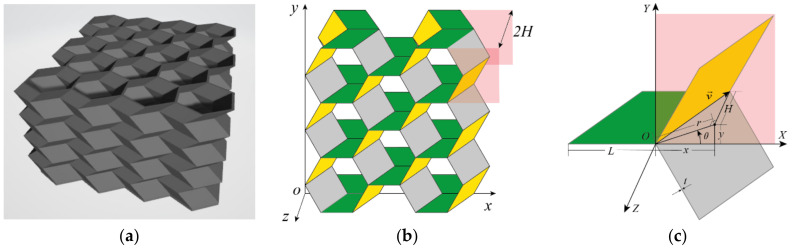
Geometric parameters of origami honeycomb structures: (**a**) overall structure, (**b**) coordinate diagram, and (**c**) geometric properties.

The origami honeycomb is folded from the conventional honeycomb, as illustrated in Figure 1, and the arris edges of the conventional honeycomb are folded in accordance with the Miura origami pattern. Hence, the origami honeycomb depicted in Figure 2b can be regarded as a combination of multiple oblique hexagonal prisms. A pair of symmetrical hexagonal prisms can be regarded as a unit cell. x and y are the in-plane coordinates, where the x-coordinate is parallel to one side of the hexagon. z is the out-of-plane coordinate, which is perpendicular to the plane where the hexagon lies. $n_{1}$, $n_{2}$, and $n_{3}$ are the number of unit cells in the x, y, and z coordinates, respectively.

The Y-cellular cell can also be considered as a unit cell, when all of the walls of the origami honeycomb are of equal thickness. The geometry of a Y-cellular cell with its characteristic parameters is shown in Figure 2c. The folding direction and length of the unit cell can be obtained from the deviation vector $\vec{v}=(x,y,H)$, where *H* represents the height of each layer of oblique prisms, and (x,y) is the offset coordinate of the corresponding point on two regular hexagons. In addition, $\vec{v}$ can also be represented by (r,θ,H), where $r=\sqrt{x^{2}+y^{2}}$ is the misalignment displacement and θ=arctan(y/x) is the misalignment angle.

Of all the parameters, only five independent ones are needed to completely define the geometry of the pattern: side length *L*, wall thickness *t*, unit height *2H*, misalignment displacement *r*, and misalignment angle θ. All other parameters can be determined by these five parameters.

## 3. Numerical Simulation of Origami Honeycomb

### 3.1. Numerical Model

**Figure 3:**
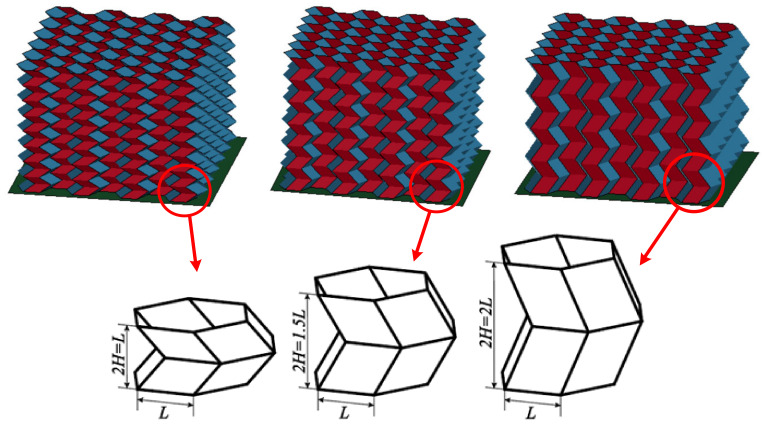
Hexagonal prismatic unit cells of origami honeycomb with different *H*.

In previous research, the energy absorption characteristics of the origami honeycomb with H=L=8.8 mm have been studied. However, as shown in Figure 3, the value of *H* affects the number of units within the unit length in the out-of-plane direction. Since the numerical models of origami honeycomb had been validated by experiments [44], the numerical modeling approach used in this paper was adopted to study the influence of other geometric parameters on the energy absorption performance of the origami honeycomb. One conventional honeycomb and six origami honeycombs were built numerically to investigate the effect of *H* on the energy absorption characteristics. The geometrical parameters of the conventional honeycomb were L=8.8 mm, t=0.5 mm, $n_{1}=4$, and $n_{2}=6$, and the height in the out-of-plane direction $H_{conventional}=70.4\;mm$. All origami honeycombs had θ=π/6, and the same L, t, $n_{1}$, and $n_{2}$ as conventional honeycomb. In addition, the values of the parameters *H* and *r* of the origami honeycomb are shown in Table 1. The number of basic units in the out-of-plane direction was determined from $n_{3}=H_{conventional}/2H$. All honeycombs have mechanical properties with Young’s modulus *E* = 69 GPa, density *ρ* = 2740 kg/m^3^, Poisson’s ratio *v* = 0.33, and yielding stress *σ* = 220 MPa.

**Figure 4:**
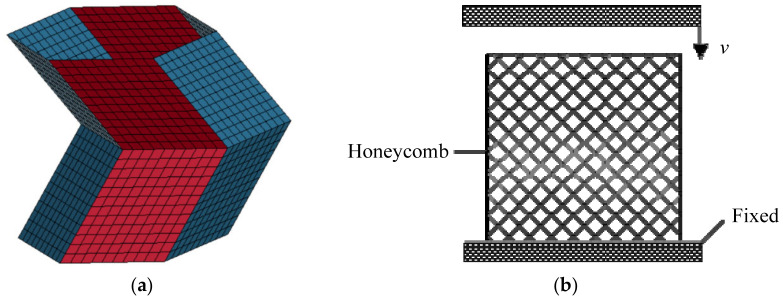
Finite element model of origami honeycomb: (**a**) mesh on unit cell, and (**b**) schematic of finite element model.

Numerical simulation models of origami honeycombs were established based on Patran, and LS-DYNA was used as an analytical solver to analyze the energy absorption characteristics of the origami honeycomb structure under compressive loads in different directions. The finite element model of a unit cell is shown in Figure 4a. We selected the elastoplastic constitutive model and applied it as plastic kinematics (MAT3). The selection of properties was homogenous and the Belytschko Tsay shell element was used. IsoMesh was used to mesh the geometry, and we used quadrilateral mesh with edge length of 0.1L. The schematic of the finite element model is illustrated in Figure 4b. In order to simulate the mechanical behavior of the origami honeycomb structure under compression load, the honeycomb was placed on a completely fixed rigid plate, and a moving rigid plate moved downward to crush the honeycomb. No constraints were imposed on the honeycomb. All of the degrees of freedom of the fixed rigid plate were fixed, but only the translational degrees of freedom of the moving plate in the vertical direction were not restricted. Self-contact was established between the external surfaces of the honeycomb structure to prevent penetration caused by the surface of the model, and surface-to-surface contact was defined between the honeycomb and each rigid plate. Friction was taken into consideration: the dynamic friction coefficient between the rigid plate and the honeycomb structure was set to 0.17, and the friction coefficient between the honeycomb cell walls was 0.1. To ensure the accuracy of simulation results, the sampling points for simulation were set to 1000.

### 3.2. Numerical Results and Analysis

**Figure 5:**
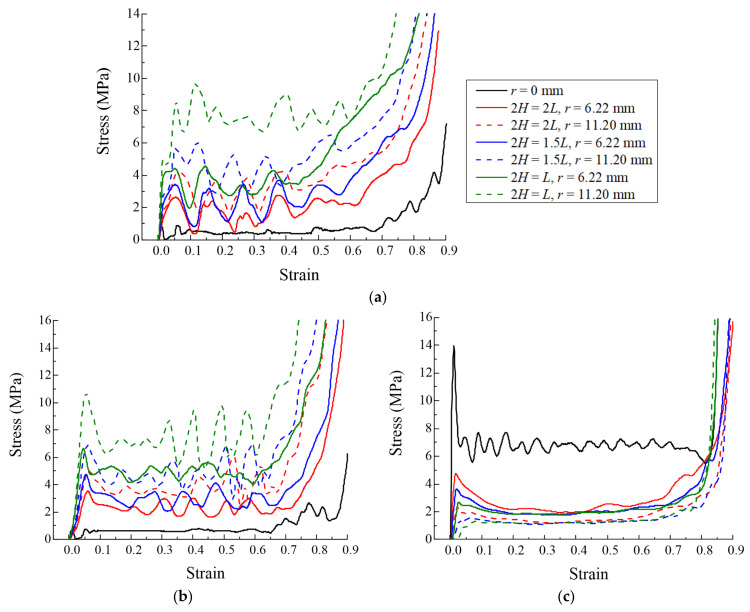
Stress versus strain curves of different models (**a**) in the in-plane direction along the y-coordinate, (**b**) in the in-plane direction along the x-coordinate, and (**c**) in the out-of-plane direction.

Seven finite element models were analyzed to investigate the energy absorption characteristics of the origami honeycomb in the in-plane and out-of-plane directions, including one conventional honeycomb and six origami honeycombs with different *H* and r values. The model parameters and simulation results are listed in Table 1. The stress–strain curves of different models are depicted in Figure 5. In the in-plane direction, as shown in Figure 5a,b, the stress on the conventional honeycomb decreases rapidly after reaching the initial peak stress value, and the mean stress along the y-coordinate and x-coordinate directions are only 0.488 and 0.582 MPa, respectively. However, the stress value of the origami honeycomb always maintains a high value, accompanied by a series of crests and troughs. When *H* remains unchanged, a larger *r* will result in a higher mean stress. This is consistent with the conclusions of previous studies [44]. On the other hand, when *r* remains unchanged, the mean stress increases as *H* decreases. A smaller *H* means that there are more origami units in the out-of-plane direction. Therefore, the energy absorption caused by the bending of the plastic hinge increases, and the mean stress of the origami honeycomb is improved. It can be seen that when *r* = 11.20 mm and *H* = *L*, the strengths in the two in-plane directions of the origami honeycomb are 5.88 and 5.92 times that of the conventional honeycomb, respectively. Furthermore, when *r* = 11.20 mm and *2H* = *L*, the strengths in the two in-plane directions of the origami honeycomb are 15.14 times and 11.92 times higher than those of the conventional honeycomb, respectively. In the out-of-plane direction, as depicted in Figure 5c, the creases of the origami honeycomb form multiple preset plastic hinges in the out-of-plane direction, thereby reducing the strength of the honeycomb in this direction. When *H* remains unchanged, a larger *r* will result in a lower mean stress. This is also consistent with the conclusions of previous studies [44]. On the other hand, when *r* remains unchanged, the mean stress decreases as *H* decreases. In conclusion, a decrease in *H* leads to an enhancement in the in-plane strength of the origami honeycomb. Therefore, this paper will focus on the *2H* = *L* origami honeycombs.

### 3.3. Deformation Mode and Mean Stress of Origami Honeycomb

To analyze the deformation mode of the 2*H* = *L* origami honeycomb in the three directions and the relationship between the deformation mode and geometric parameters of the origami honeycomb, numerical simulations were performed on the origami honeycombs with r being $(0.1,0.3,0.5,0.7,0.9)\times \sqrt{2}L\;\mathrm{mm}$ and θ being (1,2,3,4,5)×(π/12), as presented in Table 2.

**Figure 6:**
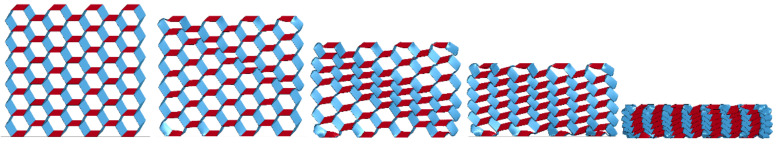
In-plane crushing deformation of origami honeycomb along the *y*-coordinate.

The deformation process of the origami honeycomb under the compression load along the *y*-coordinate is illustrated in Figure 6. In the *y*-coordinate crushing process, all adjacent cells collapse layer by layer in the same inclined direction until all cells are compressed to the dense stage. In other words, the origami honeycomb structure exhibits a layered crushing deformation pattern.

**Figure 7:**
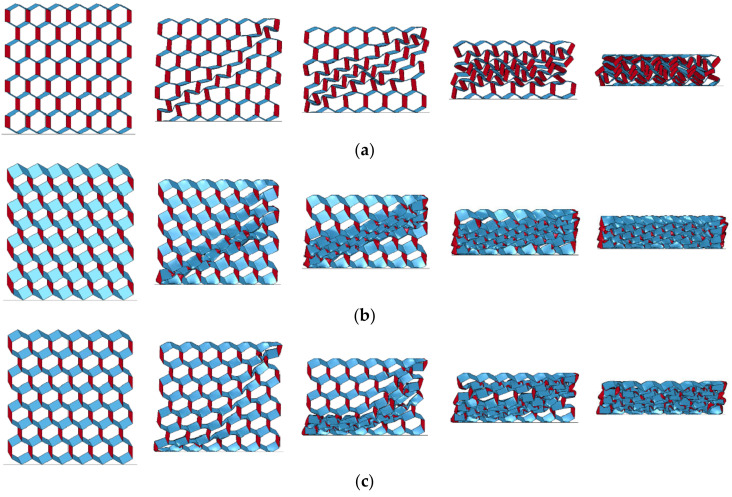
In-plane crushing deformation of origami honeycomb along the *x*-coordinate: (**a**) symmetrical deformation pattern, (**b**) one-sided deformation pattern, and (**c**) compound deformation pattern.

The deformation process of the origami honeycomb under the compression load along the x-coordinate is illustrated in Figure 7. Similar to the *y*-coordinate, in the *x*-coordinate crushing process, the origami honeycomb structure also exhibits a layered crushing deformation pattern. However, the origami honeycombs with different folding parameters produce different deformation patterns, namely, symmetrical deformation pattern (Figure 7a), one-sided deformation pattern (Figure 7b), and compound deformation pattern (Figure 7c).

**Figure 8:**
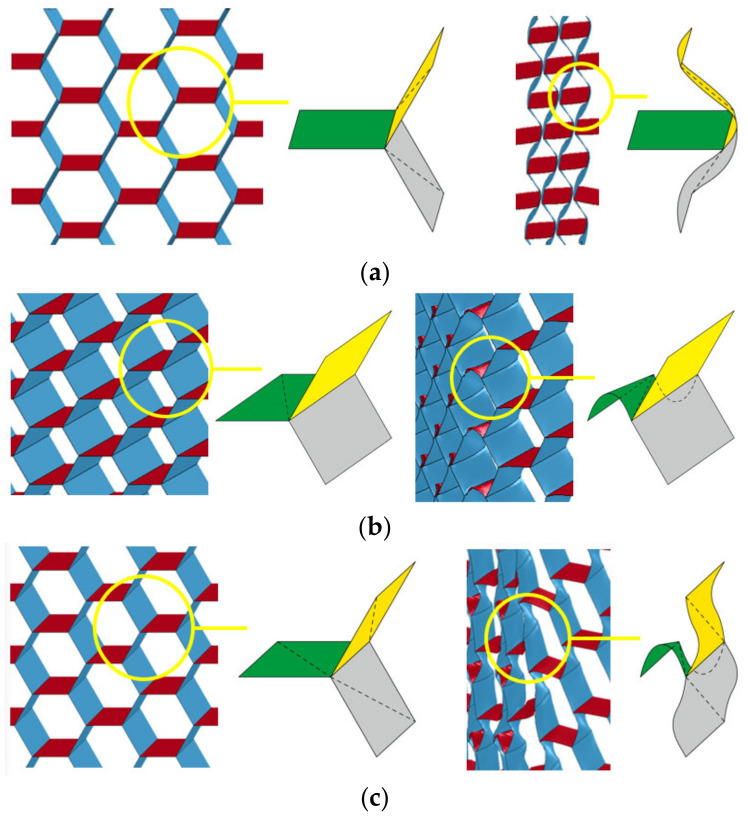
Y-cellular in (**a**) symmetric deformation pattern; (**b**) one-sided deformation pattern; and (**c**) compound deformation pattern.

The deformation of the Y-cellular cell of the origami honeycomb that produces the symmetrical deformation pattern is illustrated in Figure 8a. The middle vane of the Y-cellular cell is not significantly deformed, whereas the upper and lower vanes are significantly bent inward, and the origami honeycomb finally takes an hourglass shape. The deformation of the Y-cellular cell of the origami honeycomb that produces the one-sided deformation pattern is depicted in Figure 8b. The upper and lower vanes of the Y-cellular cell are not significantly deformed, but the middle vane is considerably deformed, and the origami honeycomb finally takes a diamond shape. For the compound deformation pattern, the deformation of the Y-cellular cell combines the characteristics of the symmetrical deformation pattern and those of the one-sided deformation pattern, and all three of the vanes exhibit large deformations, as depicted in Figure 8c.

**Figure 9:**
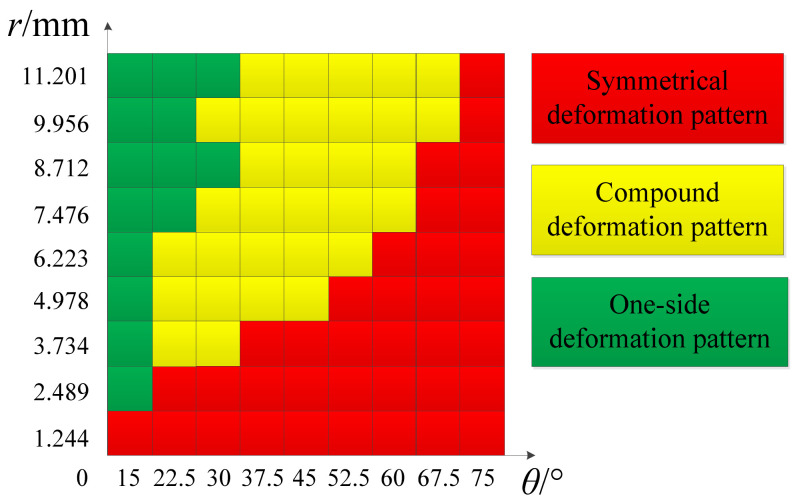
Distribution of deformation modes in the x-direction.

In order to accurately analyze the relationship between the deformation mode along the x-coordinate and the geometric parameters of the origami honeycomb, the number of models has been expanded, and the folding parameters are $r=(0.1,0.2,\cdots ,0.9)\times \sqrt{2}L\;\mathrm{mm}$ and θ=(2,3,⋯,10)×(π/24), respectively. Figure 9 shows the distribution of the deformation modes with changes in the folding parameters. When θ is relatively large, it is easy to produce a symmetrical deformation mode, and when θ remains unchanged, a lower r is more likely to produce a symmetrical deformation mode. When θ is relatively small, the one-sided deformation mode is likely to occur, and when θ remains unchanged, a higher r is more likely to produce a one-sided deformation mode. The origami honeycombs in the middle distribution zone are more likely to produce compound deformation modes.

**Figure 10:**
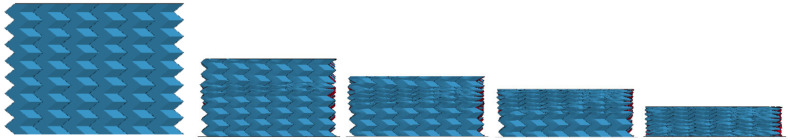
Out-of-plane crushing deformation of origami honeycomb.

The deformation process of the origami honeycomb under the compression load along the z-coordinate is depicted in Figure 10. In the out-of-plane crushing process, the origami honeycomb undergoes a uniform layered collapse along the crease.

**Figure 11:**
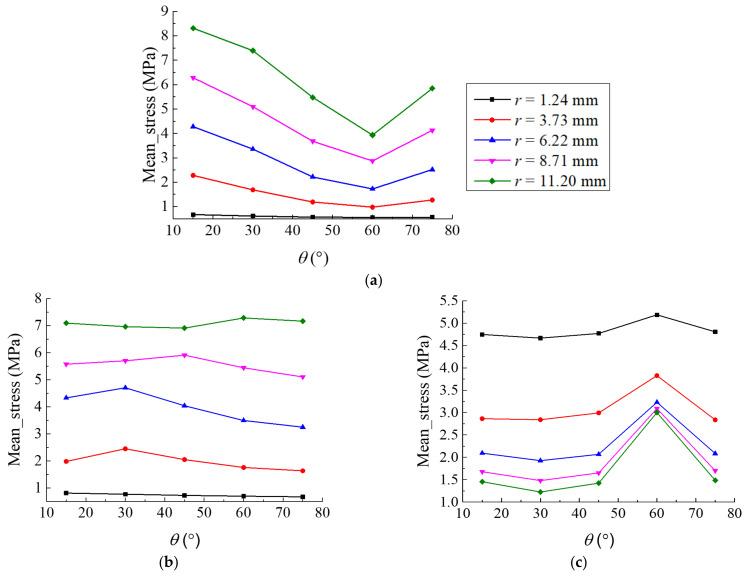
Mean stress under the compression of the origami honeycomb: (**a**) in the in-plane direction along the y-coordinate, (**b**) in the in-plane direction along the x-coordinate, and (**c**) in the out-of-plane direction.

The mean stress under the compression of the origami honeycomb is shown in Figure 11. In the *y*-coordinate direction, as shown in Figure 11a, when θ is fixed, the mean stress increases as r increases. In contrast, when r is fixed and θ increases, the mean stress first decreases and then increases beyond θ=π/3. In the x-coordinate direction, as shown in Figure 11b, the mean stress appears at “local high points” in the model of the compound deformation mode. In the z-coordinate direction, r has a significant influence on the mean stress, as shown in Figure 11c; when θ is fixed, the mean stress increases as r decreases. In contrast, when r is fixed, the mean stress does not change significantly when θ changes, but there exists a point of high value at θ=π/3.

**Figure 12:**
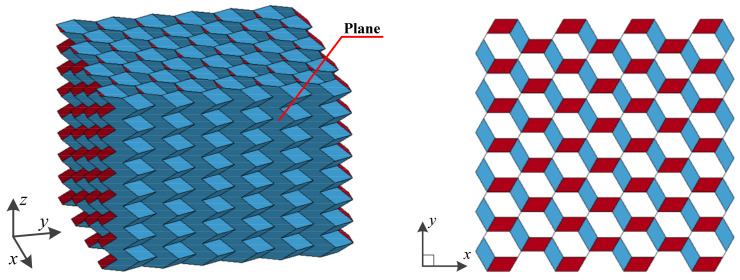
Origami honeycomb with θ=π/3

**Figure 13:**
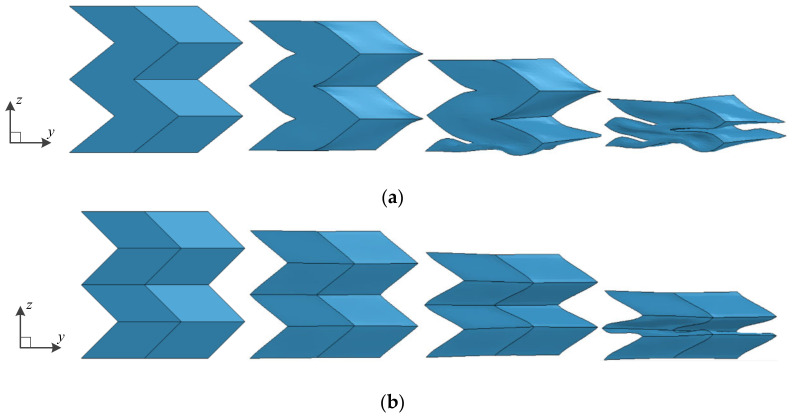
Out-of-plane crushing deformation of two Y-cellular cells when (**a**) r=6.22mm and θ=π/3, and (**b**) r=6.22mm and θ=π/4.

**Figure 14:**
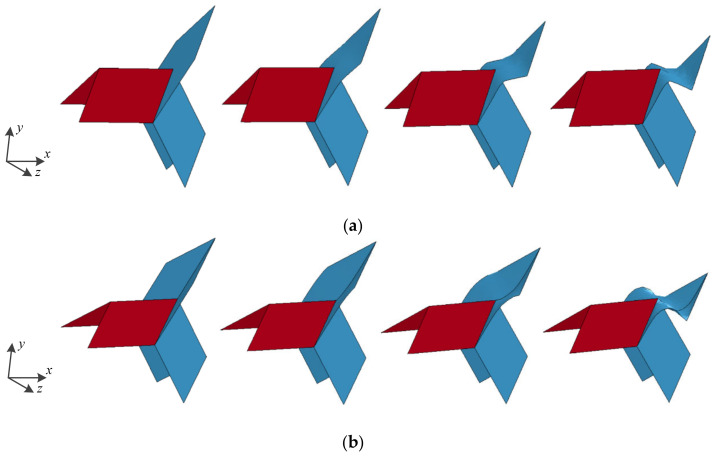
In-plane crushing deformation of Y-cellular cell along the y coordinate when (**a**) r=6.22mm and θ=π/3, and (**b**) r=6.22mm and θ=π/4.

In particular, as shown in Figure 12, one surface of the Y-cellular cell of the origami honeycomb forms a plane along the z-coordinate direction when θ=π/3. This surface produces more membrane deformation owing to the lack of crease guidance, as shown in Figure 13. Therefore, the mean stress along the z-coordinate has the highest value at θ=π/3. In contrast, the bending resistance becomes weak owing to the lack of creases when the honeycomb is subjected to the compression load along the y-coordinate, as depicted in Figure 14. Therefore, the mean stress along the y-coordinate has the lowest value at θ=π/3.

### 3.4. Response Surface Analysis of Mean Stress

The mechanical behavior of the origami honeycomb under the compression load is a highly nonlinear problem. Hence, the response surface method (RSM) is used to establish a surrogate model for the mean stress of the origami honeycomb. The polynomial response surface model can be expressed as:

(1) $$y^{r}(x)=\sum_{i=1}^{n}\beta_{i}\varphi_{i}(r,\theta ),$$

where *n* represents the number of polynomials $\varphi_{i}(r,\theta )$.

The polynomial coefficient, $\beta =\left(\beta_{1},\beta_{2},\ldots \beta_{n}\right)$, is calculated as:

(2) $$\beta ={\left(\phi^{T}\phi \right)}^{-1}(\phi^{T}y),$$

where matrix ϕ is expressed as:

(3) $$\phi =\left[\begin{array}{ccccc} \varphi_{1}(x^{\left(1\right)}) & \cdot & \cdot & \cdot & \varphi_{n}(x^{\left(1\right)})\\ \cdot & \cdot & \cdot & \cdot & \cdot \\ \cdot & \cdot & \cdot & \cdot & \cdot \\ \cdot & \cdot & \cdot & \cdot & \cdot \\ \varphi_{1}(x^{\left(m\right)}) & \cdot & \cdot & \cdot & \varphi_{n}(x^{\left(m\right)}) \end{array}\right],$$

and *m* is the number of simulation sample points.

According to the simulation results in Table 2 and Equations (2) and (3), the fourth-order functions of the mean stress of the origami honeycomb structure can be expressed as follows:

(4) $$\begin{array}{l} \sigma_{y}=1.50194855+0.40279530r-10.19131850\theta +\\ 0.03701878r^{2}+0.94585439r\theta +22.04644451\theta^{2}-\\ 0.00413141r^{3}+0.09196371r^{2}\theta -3.60609232r\theta^{2}-\\ 16.30285578\theta^{3}+0.00014203r^{4}-0.00105082r^{3}\theta -\\ 0.03987406r^{2}\theta^{2}+2.01100266r\theta^{3}+3.76438416\theta^{4}. \end{array}$$

(5) $$\begin{array}{l} \sigma_{x}=-0.45686955-0.78547347r+9.71297631\theta +\\ 0.43417300r^{2}+0.31995493r\theta -18.72172149\theta^{2}-\\ 0.04595767r^{3}-0.08727593r^{2}\theta +0.06838025r\theta^{2}+\\ 13.36833220\theta^{3}+0.00164433r^{4}+0.00092423r^{3}\theta +\\ 0.07139163r^{2}\theta^{2}-0.40113330r\theta^{3}-2.91056526\theta^{4}. \end{array}$$

(6) $$\begin{array}{l} \sigma_{z}=0.58643065-1.18227276r+42.24938868\theta +\\ 0.18221886r^{2}-1.47361366r\theta -103.15049064\theta^{2}-\\ 0.01113123r^{3}-0.01555113r^{2}\theta +2.49953971r\theta^{2}+\\ 100.11976863\theta^{3}+0.00028722r^{4}-0.00051099r^{3}\theta +\\ 0.01538684r^{2}\theta^{2}-1.17199225r\theta^{3}-33.18735860\theta^{4}. \end{array}$$

**Figure 15:**
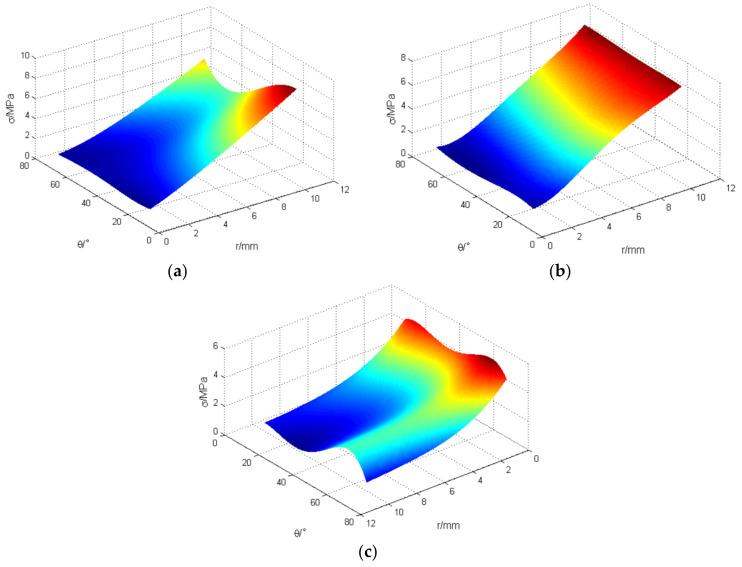
Response surfaces of mean stress for origami honeycomb: (**a**) in the in-plane direction along the *y*-coordinate, (**b**) in the in-plane direction along the *x*-coordinate, and (**c**) in the out-of-plane direction.

The response surfaces of the mean stress of the origami honeycomb structure are plotted in Figure 15. The figure reveals the influences of the displacement *r* and the misalignment angle θ on the mean stress of the origami honeycomb structure in a visual form.

The approximate solutions of the mean stress calculated using the surrogate models are listed in Table 2. Apparently, the surrogate model established by the RSM has errors. It is necessary to use the root mean square error (RMSE), the coefficient of multiple determination ($R^{2}$), and the adjusted coefficient of multiple determination ($R^{2}{}_{adj}$) to evaluate the accuracy of the RSM model [48,49].

(7) $$RMSE=\sqrt{\frac{\sum_{i-1}^{m}{\left(y(x_{i})-y(x_{i}^{r})\right)}^{2}}{m-n-1}},$$

(8) $$R^{2}=1-\frac{\sum_{i-1}^{m}{\left(y(x_{i})-y(x_{i}^{r})\right)}^{2}}{\sum_{i-1}^{m}{\left(y(x_{i})-{\bar{y}}_{i}\right)}^{2}}$$

(9) $$R_{adj}^{2}=1-\frac{m-1}{n-1}(1-R^{2})$$

where $y(x_{i})$ is the finite element simulation value, $y(x_{i}^{r})$ is the surrogate model approximate solution, and ${\bar{y}}_{i}$ is the average of the finite element analysis results for sample point *i*.

Table 3 shows the evaluation value of the approximate function of the origami honeycomb structure. $R^{2}$ and $R^{2}{}_{adj}$ in the table are very close to 1, and RMSE is close to 0. It can be proved that the established mathematical model has sufficient calculation accuracy.

## 4. Approximate Isotropy Analysis of Origami Honeycomb

Based on the research results in the previous section, the relationship between the energy absorption strength in the three directions and the folding parameters (r, θ) of the origami honeycomb can be established. To analyze the three-directional mean stress of the origami honeycomb under different folding parameters, the approximate isotropic coefficient *k* of the origami honeycomb is defined as:

(10) $$k=\frac{\sigma_{\max}-\sigma_{\min}}{\sigma_{\max}},$$

where $\sigma_{\max}$ and $\sigma_{\min}$ are the highest and lowest mean stresses in the three directions of the origami honeycomb.

**Figure 16:**
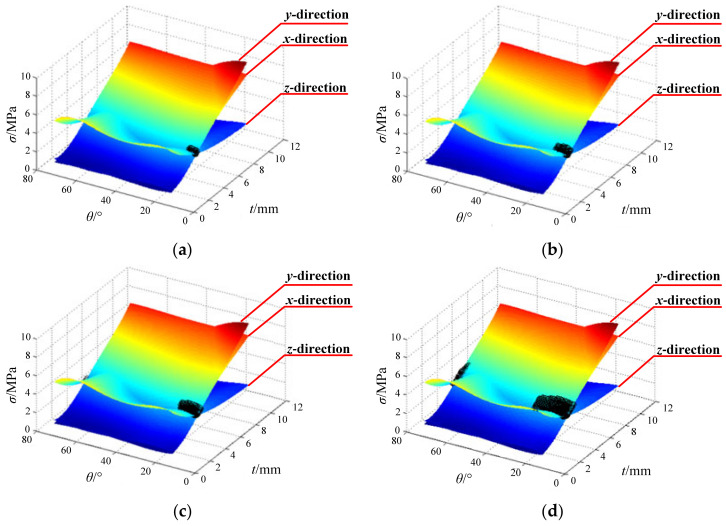
Curved surface diagram of origami honeycomb cushioning capacity when (**a**) *k* ≤ 0.1, (**b**) *k* ≤ 0.15, (**c**) *k* ≤ 0.2, and (**d**) *k* ≤ 0.3.

When the structure has the same mean stress in the *x*, *y*, and *z* directions, its approximate isotropy coefficient value *k* = 0. Therefore, we define this structure as an approximately isotropic structure. In this way, the degree of approximate isotropy of the origami honeycomb can be determined according to the value of *k*, and its ability to resist omnidirectional impact can be understood more closely. In other words, when the folding parameters are given, the surrogate model established by the RSM can be used to calculate the approximate isotropic coefficient *k* of the origami honeycomb. Conversely, when the range of *k* is given, the folding parameters of the corresponding origami honeycomb can be filtered out using the surrogate model. As shown in Figure 16, the origami honeycombs with different ranges of *k* are marked with black on the mean stress response surface, and Table 4 lists 10 origami honeycombs with 0.15 ≤ *k* ≤ 0.35.

## 5. Experiment

### 5.1. Experimental Equipment and Specimen

**Figure 17:**
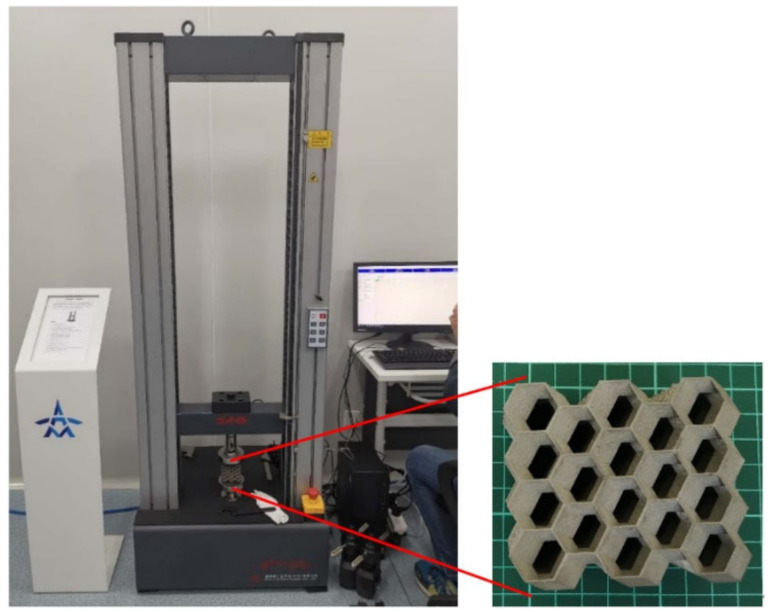
Experimental equipment and specimen.

To verify the accuracy of the simulation model in this study and the effectiveness of the isotropic design method for the origami honeycomb, the universal impact testing apparatus was used to perform the compression test on the origami honeycomb specimen, as shown in Figure 17. Three origami honeycombs satisfying 0.1 ≤ *k* < 0.2, 0.2 ≤ *k* < 0.3 and *k* ≥ 0.3 were randomly selected using the approximate isotropic evaluation system in Section 4. The specific parameters of the three honeycombs used to make the experimental specimens are listed in Table 5. In the previous work, we fabricated origami honeycombs made of aluminum alloys and conducted compression tests. The experimental results show that the aluminum alloy origami honeycomb has great brittleness, and its buffer performance fluctuates greatly. Therefore, in this paper, stainless steel was used to make origami honeycomb specimens. Additionally, all specimens were fabricated using a 3D printer. The stainless steel material has mechanical properties with Young’s modulus *E* = 139.5 GPa, density *ρ* = 7980 kg/m^3^, Poisson’s ratio *v* = 0.33, and yielding stress *σ* = 456 MPa.

### 5.2. Experimental Results

**Figure 18:**
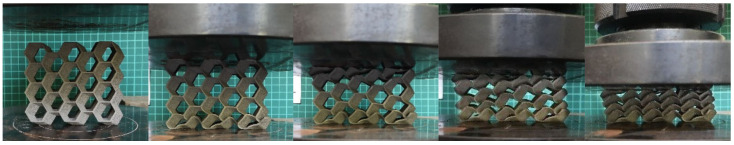
In-plane compression of origami honeycomb specimen along the *y*-coordinate.

**Figure 19:**
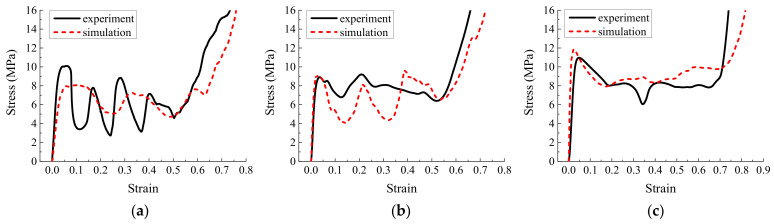
Simulation stress–strain results and experimental measurements of Specimen 1 (**a**) in the in-plane direction along the *y*-coordinate, (**b**) in the in-plane direction along the *x*-coordinate, and (**c**) in the out-of-plane direction.

**Figure 20:**
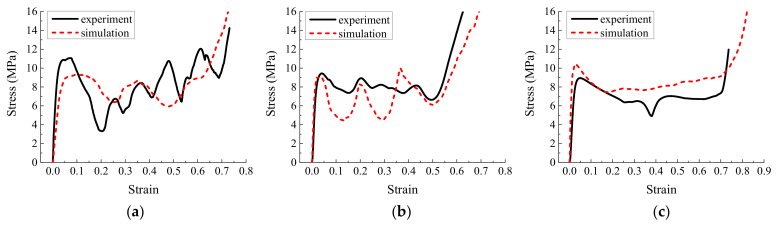
Simulation stress–strain results and experimental measurements of Specimen 2 (**a**) in the in-plane direction along the *y*-coordinate, (**b**) in the in-plane direction along the *x*-coordinate, and (**c**) in the out-of-plane direction.

**Figure 21:**
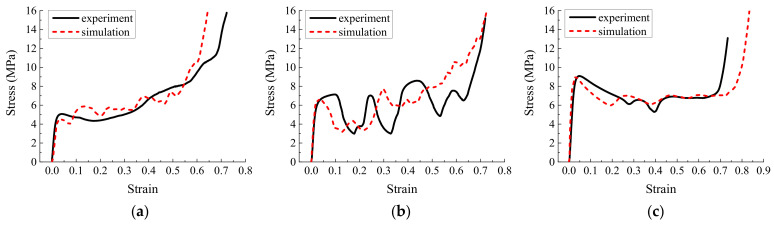
Simulation stress–strain results and experimental measurements of Specimen 3 (**a**) in the in-plane direction along the *y*-coordinate, (**b**) in the in-plane direction along the *x*-coordinate, and (**c**) in the out-of-plane direction.

In addition to the compression experiment on the specimen (Figure 18), we simulated the origami honeycomb with the same geometric structure and mechanical properties. The comparison of the simulation and experimental results is depicted in Figure 19, Figure 20 and Figure 21. It can be observed from the experimental results that the stress curves are similar to those in the simulation in terms of both magnitude and trend. The mean stress values for each model are listed in Table 6. It can be seen that the experimental mean stress of Specimen 1 in the y, x, and z directions is 7.34 MPa, 5.74 MPa, and 8.25 MPa, respectively. Therefore, according to Equation (10), the approximate isotropic coefficient of Specimen 1 can be calculated as 0.3. The simulated mean stress of Specimen 1 in the y, x, and z directions is 6.41 MPa, 6.21 MPa, and 8.92 MPa, respectively. The maximum relative error between simulation results and experimental results is −14.51%. Similarly, the experimental mean stress of Specimen 2 in three directions is 7.41 Mpa, 8.26 Mpa, and 7.13 MPa, and the approximate isotropic coefficient is 0.14. The simulated mean stress is 6.63 MPa, 7.78 MPa, and 8.32 MPa. The maximum relative error is 14.30%. Finally, the experimental mean stress of Specimen 3 in three directions is 6.01 MPa, 5.71 MPa, and 6.96 MPa, and the approximate isotropic coefficient is 0.18. The simulated mean stress is 6.1 MPa, 5.96 MPa, and 6.85 MPa. The maximum relative error is 4.19%. In summary, the experimental results verify the accuracy of the simulation and the effectiveness of the design method of approximately isotropic origami honeycomb.

## 6. Results

In this study, we proposed an origami honeycomb structure based on the Miura origami pattern, which can significantly improve the in-plane strength of the honeycomb structure. Based on this characteristic of the origami honeycomb, we designed its geometrical parameters to obtain a structure with approximate isotropic properties in three directions.

Numerical analyses were performed to simulate the in-plane and out-of-plane crushing of the origami honeycomb. The effects of geometric parameters such as unit cell height H, misalignment displacement r, and misalignment angle θ on the energy absorption characteristics of the origami honeycomb structure were analyzed. At the same time, the deformation modes of the origami honeycomb in three directions were obtained, and the in-plane deformation along the *x*-coordinate was related to the folding parameters of the origami honeycomb. In addition, the RSM was used to establish a surrogate model of the mean stress for the origami honeycomb with a high fitting accuracy. Therefore, the surrogate model established in this study can be applied to a wider range of origami honeycombs.

The approximate isotropy of the origami honeycomb was defined, and the concept of the approximate isotropic coefficient *k* was proposed such that the degree of approximate isotropy of the origami honeycomb could be determined.

Finally, three approximately isotropic origami honeycombs were designed, and finite element simulations and compression experiments were performed on them. The results indicated a favorable correlation between the simulation and the experiment, and the relative error of mean stress was in the range of −14.51~14.3%. Additionally, this also proves the correctness of the approximate isotropic honeycomb design method in this paper.

## Figures and Tables

**Table 1:** Summary of numerical modeling results.

Model	H	r/mm	σ_y_/MPa	Relative Value	σ_x_/MPa	Relative Value	σ_z_/MPa	Relative Value
0-1		0	0.488	1	0.582	1	6.784	1
1-1	*2H* = *2L*	6.22254	1.540	3.16	2.229	3.83	2.458	0.36
1-2	*2H* = *2L*	11.20057	2.867	5.88	3.462	5.95	1.436	0.21
2-1	*2H* = *1.5L*	6.22254	2.173	4.45	2.895	4.97	2.128	0.31
2-2	*2H* = *1.5L*	11.20057	4.193	8.59	4.675	8.03	1.247	0.18
3-1	*2H* = *L*	6.22254	3.356	6.88	4.705	8.08	1.927	0.28
3-2	*2H* = *L*	11.20057	7.389	15.14	6.963	11.96	1.226	0.18

**Table 2:** Comparison of response surface approximate solution and simulation values of mean stress for origami honeycomb structures.

Model	H	r/mm	Simulation Value			RSM Approximate Solution		
			*σ_y_*/MPa	*σ_x_*/MPa	*σ_z_*/MPa	*σ^r^_y_*/MPa	*σ^r^_x_*/MPa	*σ^r^_z_*/MPa
1	1.244508	π/12	0.672	0.813	4.746	0.699	0.713	4.710
2	1.244508	π/6	0.613	0.771	4.664	0.505	0.927	4.773
3	1.244508	π/4	0.568	0.727	4.768	0.623	0.689	4.575
4	1.244508	π/3	0.557	0.698	5.184	0.627	0.564	5.384
5	1.244508	5π/12	0.561	0.675	4.805	0.517	0.790	4.725
6	3.733524	π/12	2.280	1.981	2.864	2.322	2.142	2.892
7	3.733524	π/6	1.686	2.451	2.841	1.711	2.385	2.890
8	3.733524	π/4	1.189	2.050	2.993	1.191	2.106	2.877
9	3.733524	π/3	0.978	1.759	3.826	0.875	1.762	3.808
10	3.733524	5π/12	1.270	1.635	2.838	1.304	1.482	2.895
11	6.22254	π/12	4.277	4.330	2.094	4.215	4.214	2.051
12	6.22254	π/6	3.356	4.705	1.927	3.345	4.418	1.953
13	6.22254	π/4	2.216	4.041	2.067	2.277	4.151	2.123
14	6.22254	π/3	1.723	3.495	3.230	1.666	3.762	3.198
15	6.22254	5π/12	2.518	3.248	2.085	2.588	3.274	2.078
16	8.711556	π/12	6.282	5.576	1.679	6.231	5.728	1.674
17	8.711556	π/6	5.094	5.702	1.481	5.235	5.848	1.439
18	8.711556	π/4	3.678	5.909	1.652	3.686	5.670	1.773
19	8.711556	π/3	2.871	5.443	3.090	2.776	5.434	3.004
20	8.711556	5π/12	4.126	5.105	1.701	4.121	5.055	1.714
21	11.20057	π/12	8.313	7.094	1.456	8.356	6.999	1.511
22	11.20057	π/6	7.389	6.963	1.226	7.342	7.013	1.084
23	11.20057	π/4	5.477	6.910	1.424	5.351	7.020	1.556
24	11.20057	π/3	3.930	7.285	3.002	4.115	7.157	2.939
25	11.20057	5π/12	5.842	7.167	1.486	5.788	7.230	1.503

**Table 3:** Origami honeycomb structure approximation function evaluation values.

	RMSE	$R^{2}$	$R_{adj}^{2}$
*σ_y_*	0.1251	0.9989	0.9973
*σ_x_*	0.2240	0.9967	0.9920
*σ_z_*	0.1462	0.9949	0.9878

**Table 4:** Ten examples of origami honeycomb with 0.15 ≤ *k* ≤ 0.35.

Model	*r*/mm	*θ*/rad	Approximate Solution			*k*
			*σ^r^_y_*/MPa	*σ^r^_x_*/MPa	*σ^r^_z_*/MPa	
1	3.9	1/9π	2.22	2.46	3.02	0.27
2	4.0	4/45π	2.79	2.59	4.00	0.35
3	4.2	1/9π	2.43	2.72	2.88	0.16
4	4.3	1/6π	2.05	2.86	2.62	0.28
5	4.4	2/15π	2.39	2.95	2.85	0.19
6	4.5	1/9π	2.80	3.35	3.61	0.23
7	4.6	1/8π	2.60	3.11	2.69	0.16
8	4.7	1/10π	2.89	3.09	2.63	0.15
9	4.8	1/9π	2.87	3.23	2.63	0.19
10	5	5/12π	2.74	2.68	3.20	0.16

**Table 5:** Geometric parameters of origami honeycomb used in experiments.

Model	*r*/mm	*θ*/rad	*n_x_*	*n_y_*	*n_z_*	*H*/mm	*L*/mm	*t*/mm
Specimen 1	4	4/45π	3	4	4	4.4	8.8	0.5
Specimen 2	4.5	1/9π	3	4	4	4.4	8.8	0.5
Specimen 3	5	5/12π	3	4	4	4.4	8.8	0.5

**Table 6:** Comparison of simulation results and experimental results.

		*σ^r^_y_*/MPa	*σ^r^_x_*/MPa	*σ^r^_z_*/MPa	*k*
Specimen 1	Simulation value	6.41	6.21	8.92	0.30
	Experimental value	7.34	5.74	8.25	0.30
	Relative error (%)	−14.51	7.57	7.51	
Specimen 2	Simulation value	6.63	7.78	8.32	0.21
	Experimental value	7.41	8.26	7.13	0.14
	Relative error (%)	−11.76	−6.17	14.30	
Specimen 3	Simulation value	6.1	5.96	6.85	0.13
	Experimental value	6.01	5.71	6.96	0.18
	Relative error (%)	1.48	4.19	−1.61	

## Data Availability

The data presented in this study are available upon request from the corresponding author.
